# Effects of probiotics on gastrointestinal symptoms, anthropometric measurements, and breastfeeding duration in infants with colic: a randomized control trial

**DOI:** 10.1590/1516-3180.2023.0069.R1.31052023

**Published:** 2024-04-22

**Authors:** Aysu Yıldız Karaahmet, Gülümser Dolgun, Metehan Özen

**Affiliations:** I PhD. Assistant Professor, Department of Midwifery, Faculty of Health Science, Halic University, Istanbul, Turkiye.; II PhD. Professor, Department of Midwifery, Faculty of Health Science, Istanbul University-Cerrahpasa, Istanbul, Turkiye.; III MD. Professor, Department of Child Health and Diseases, Faculty of Medicine, Acıbadem University, Istanbul, Turkiye.

**Keywords:** Colic, Crying, Probiotics, Maternal nutritional physiological phenomena, Fussing, Excessive crying, Infants, Complementary and alternative medicine

## Abstract

**BACKGROUND:**

Infantile colic has a multifactorial etiology. Recent studies have suggested that probiotics may be effective in its management.

**OBJECTIVE:**

This study was carried out to evaluate the effect of the Actiregularis strain (5×10^6^ cfu\ml) included in maternal nutrition on gastrointestinal problems, growth development, and breastfeeding outcomes in infantile colic.

**DESIGN AND SETTING:**

The study was a randomized controlled trial conducted in the neonatal outpatient clinic of a training and research hospital in Turkey.

**METHODS:**

A probiotic drink containing the Actiregularis (5×10^6^ cfu\ml) strain was added to the diet of mothers in the probiotics group once daily for 15 consecutive days. Data were collected for each infant’s 0th (birth), 1st, 4th, and 6th months.

**RESULTS:**

Infants whose mothers were administered Actiregularis for 15 days had decreased crying intensity (P = 0.000). When the difference in breastfeeding rates between the groups was significant at the 4th and 6th months (P = 0.044; P = 0.035). There was no difference in anthropometric values except the babies’ weights at the 6th month. (P < 0.001).

**CONCLUSION:**

Infants treated with Actiregularis, which was added to their mothers’ diet for 15 days, showed a decrease in the frequency of crying, and the difference in breastfeeding rates between the groups was significant at the 4th and 6th months. There was no difference in anthropometric values except the babies’ weights at the 6th month.

**CLINICAL TRIALS REGISTRATION:**

NCT04374955 (https://clinicaltrials.gov/ct2/show/).

## INTRODUCTION

Infantile colic was first described by Wessel et al.^1^ in 1954 as “crying attacks that last three hours a day, three days a week, and continue for three weeks.” Infantile colic is a benign newborn problem characterized by self-limiting, unpreventable crying attacks that affect 25% of babies in the first three months of life. It is a very troublesome process for parents and babies.^2^ It is associated with short- and long-term negative outcomes such as postpartum maternal depression, early cessation of breastfeeding, parental guilt and frustration, shaken baby syndrome, multiple doctor visits, medication use, growth and development problems, allergies, and behavior and sleep problems.^3,4^

The pathogenesis of infantile colic and the conditions that cause its disorder have not yet been elucidated. Some studies have suggested that maternal malnutrition, consumption of foods containing allergens, and lactase deficiency in infants are responsible.^5^ In recent years, it has been thought that the gastrointestinal tract flora may play a role in infantile colic.^6,7^ It has been suggested that deteriorated intestinal flora (dysbiont), which is thought to be responsible for infantile colic, can be improved by probiotics and ameliorated colic pain in infants.^8,9^ Probiotics play an important role in changing the flora, possibly by causing bacterial diversity in the gastrointestinal tract via the gut-brain axis.^8^ Some studies have suggested the valuable effects of maternal intake of probiotics during the perinatal or postpartum period to change the intestinal flora of infants in the prevention of infantile colic.^8,10,11^ Many studies have been carried out to evaluate the use of probiotic supplements in the treatment of infantile colic and have shown their effectiveness on crying duration, gastrointestinal problems, growth and development parameters, and breastfeeding duration of the baby.^10,12^ However, in infantile colic studies, probiotic products are generally included in infant nutrition, while studies on including probiotic products in maternal nutrition are almost non-existent.^13,14^

## OBJECTIVE

This randomized controlled trial was carried out to evaluate the effect of the Actiregularis strain (5×10^6^ cfu\ml) included in maternal nutrition on gastrointestinal problems, growth development, and breastfeeding outcomes in infantile colic.

## METHOD

### Study population and design

Study is the only blind, randomized, controlled clinical trial conducted to evaluate the effect of maternal probiotic administration on gastrointestinal issues, growth-development, and breastfeeding outcomes in infants with colic. The study was conducted in the children outpatient clinic of a training and research hospital in Istanbul, Turkey, between August 2020 and February 2021. Inclusion criteria required to comply with the study protocol were as follows: (a) age less than 60 days, (b) vaginal delivery, (c) breastfeeding more than 8–10 times a day (more than 50%) and (d) being born at term (e) diagnosis of colic (one week before the start of the study, and the crying duration lasting more than three hours a day for at least three days in a week). Exclusion criteria were as follows: (a) presence of major acute or chronic diseases in the mother and infant, (b) gastrointestinal diseases and gastro-esophageal reflux, (c) maternal use of probiotics/antibiotics one week before or during randomization, (d) gastrointestinal malformations, (e) presence of maternal depression. The exclusion criteria also included not using the probiotic product twice simultaneously, using medication for infantile colic, stopping breastfeeding during supplementation, and using formula.

This study was approved by the Training and Research Hospital Clinical Research Ethics Committee (10.06.2020. Ethics Committee No: 2020-85), and institutional permission was obtained from the same institution. Written informed consent was obtained from the mothers accordingly.

When the infants who presented to the clinic with a complaint of crying were referred by a pediatrician with a diagnosis of infantile colic and fulfilled all criteria for registration, the parents were given a 7-day questionnaire that assessed crying duration, sleep, wakefulness, and feeding. Seven days later, an appointment was made for diagnosis; infants and their mothers who met the criteria for colic were enrolled in the study (150 mothers and infants who were diagnosed).

**Figure 1:**
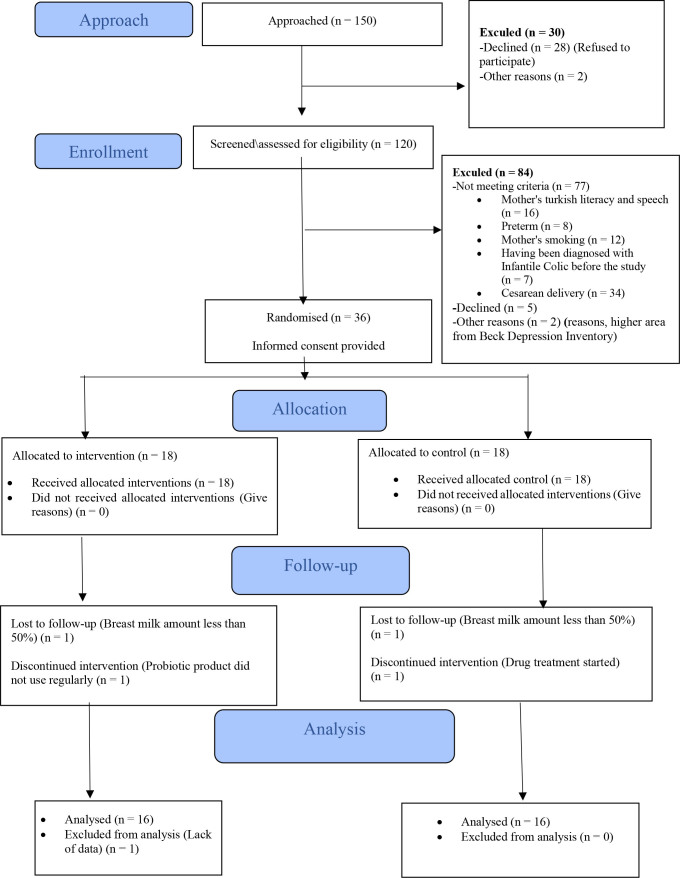
Flowchart of patients considered for the trial of probiotics supplementation for mothers of infants with colic.

The minimum sample size was calculated using the power analysis statistical software package version 9.3 (SAS Institute, Cary, North Carolina, United States) based on a 50% reduction in daily crying time in infants in the intervention group (those administered a probiotic product).^13^ According to this calculation, it was determined that 30 mother-infant pairs should be included in this study, with alpha level of 0.05 and 80% power at 95% confidence interval. However, considering case losses, the sample size was set at 36 mother-infant pairs. At the end of the data collection process, five mother-infant pairs were excluded for various reasons (Figure 1), and 31 mother-infant pairs were finally included in the study sample.

All mothers included in the study were randomized into two groups: probiotic and control groups. Randomization was performed in a 1:1 ratio to groups A and B according to a randomization list created by a specific software (www.randomizer.org). An independent researcher prepared the randomization program. According to a lottery method, the patients were assigned to probiotics group (A) or control group (B). Randomization codes were kept confidential until all data were analyzed (Figure 1).

All participants, as well as statisticians who evaluated the results of the research, were blinded to the group assignment. Data were collected by a researcher who was not blinded to the study. To avoid bias, the researcher did not participate in any statistical analyses.

Mothers assigned to the probiotics group were instructed to drink a probiotic product containing one can (80 ml) of Actiregularis (5×10^6^ cfu\ml) strain once a day for 15 days, directly by mouth, preferably in the morning on a full stomach, and without consuming any liquid, including food and water, for an hour. Instructions for storing and handling the product were provided in accordance with the manufacturer’s instructions. The mothers were given a diary and taught how to record their administration of the daily dose of the study product as well as the infant’s severity of crying and the frequency of stools daily. The mothers were asked to return the used bottles to the researcher after 15 days. Although maternal diet affects the frequency of colic, no special dietary restrictions were recommended during breastfeeding, except to avoid any commercial products containing probiotics.^15^ The mothers were also instructed to avoid other methods of treating infantile colic. The mothers in the control group received routine care (behavioral therapy) from the institution. Prebiotics were given free of charge to mothers.

### Analysis of symptoms of infantile colic

A baby diary form was given to each parent to record gastrointestinal events, such as the baby’s feeding schedule, daily fuss/crying attacks, frequency of uncontrollable crying per day, intensity of daily crying, number of daily bowel movements, and consistency of stools. The frequency of crying was reported as “increased” or “decreased,” and was evaluated using the same terms as the previous day, while crying intensity was scored between 0 and 10 points.

At the time of registration (day 0), a pediatric medical examination was performed, and the following information was collected: (1) gestational age, (2) mode of birth, (3) birth weight, (4) anthropometric data at admission, (5) family history of gastrointestinal disease, and (6) family history of atopy.

Follow-up visits were planned at 15 days, 4 months, and 6 months after the start of the application of the study product. At the end of the 15th day of the study, all diaries were collected. Study analysis and data entry were performed independently by the researchers, both of whom were blinded to the treatment allocation.

### Statistical analysis

The primary outcome measures were the ratio of those who responded and those who did not respond to treatment in improving colic symptoms. Success rate was defined as a 50% reduction in the average daily crying frequency and intensity, expressed as frequency and severity.

The secondary outcome measures were the baby’s (1) daily defecation consistency, (2) anthropometric evaluations (6-month outcomes), and (3) breastfeeding status (6-month results).

Data from initial visit forms, logs, and the results of the analysis of fecal samples were reported in a database created using Google Drive software (Google ILC, Mountain View, California, United States). The data obtained in this study were analyzed using SPSS (IBM, Armonk, NY, USA) for Windows 25.0.

Quantitative variables with a normal distribution were compared using an independent samples t-test. The Mann–Whitney U test was used for variables without a normal distribution, a comparison was carried out using the analysis of variance for repeated measurements when the variables had a normal distribution. In addition, the Friedman test was applied when the variables did not have a normal distribution, and the Bonferroni test was used to find the time that made the difference in the case where a difference was found. The ratios were compared using the χ^2^ test or the Fisher’s exact test as appropriate. For all tests, P < 0.05 value was considered significant.

## RESULTS

For eligibility, 36 mothers and their infants were enrolled and randomized into the control (n = 18) or probiotic (n = 18) group. After 15 days of follow-up, failure was recorded in five infants; therefore, 31 infants (16 in the control group and 15 in the probiotics group) completed the study (Figure 1). No significant differences were observed in age, sex, birth weight, and diet (Table 1).

**Table 1:** Distribution of socio-demographic and obstetric characteristics of mothers in the probiotics and control groups (n = 31)

		Probiotics		Control		Test Value	P
		n	%	n	%		
Mother’s age	24 years and under	7	46.7	8	50.0	0.034**	0.853
25.58 ± 5.04	25 years and older	8	53.3	8	50.0		
Mother’s weight	9 pounds and less	7	46.7	8	50.0	0.034**	0.569
10.35 ± 5.90	10 pounds and more	8	53.3	8	50.0		
Postnatal age of the baby	Day	30.20 ± 2.21		29.56 ± 1.82		0.878**	0.387
Gestational age	Week	39.32 ± 0.87		39.10 ± 1.16		0.591**	0.559
Sex of the baby	Girl	10	66.7	0.059**	0.553	0.059**	0.553
	Boy	5	33.3	6	37.5		
**Sum**		**15**	**100.0**	**16**	**100.0**		

*P < 0.05;

**χ= Chi-square analysis.

Initially, the average crying times of the two groups were similar. On day 15, a higher and statistically significant treatment success rate was observed in the probiotics group compared with the control group (Table 2). There was a statistically significant difference between the groups in the frequency of stools on the second, third, sixth, eighth, tenth, and fourteenth days (P < 0.05) (Table 3).

**Table 2:** Distribution of crying intensity of infants in the probiotics and control groups (n = 31)

		$\bar{X}$ ± SD [Min-Max]	Test value	P
1	Intervention	9.93 ± 0.26 [9.00–10.00]	112.000**	0.770
	Control	10.00 ± 0.00[10.00–10.00]		
2	Intervention	9.87 ± 0.35 [9.00–10.00]	111.500**	0.740
	Control	9.94 ± 0.25[9.00–10.00]		
3	Intervention	9.73 ± 0.46[9.00–10.00]	95.500**	0.338
	Control	9.94 ± 0.25[9.00–10.00]		
4	Intervention	9.33 ± 0.49[9.00–10.00]	62.500**	**0.021** *
	Control	9.81 ± 0.40[9.00–10.00]		
5	Intervention	9.00 ± 0.85[8.00–10.00]	70.00**	**0.049** *
	Control	9.63 ± 0.62[8.00–10.00]		
6	Intervention	8.60 ± 0.83[7.00–10.00]	39.000**	**0.001** *
	Control	9.63 ± 0.62[8.00–10.00]		
7	Intervention	7.93 ± 1.03[7.00–10.00]	34.000**	**0.000** *
	Control	9.38 ± 0.62[8.00–10.00]		
8	Intervention	7.20 ± 1.01[6.00–9.00]	13.000**	**0.000** *
	Control	9.13 ± 0.62[8.00–10.00]		
9	Intervention	6.80 ± 1.15[5.00–8.00]	9.000**	**0.000** *
	Control	9.06 ± 0.68[8.00–10.00]		
10	Intervention	6.27 ± 1.22[5.00–8.00]	9.500**	**0.000** *
	Control	8.94 ± 0.85[7.00–10.00]		
11	Intervention	5.60 ± 0.99[4.00–7.00]	3.000**	**0.000** *
	Control	8.50 ± 0.82[7.00–10.00]		
12	Intervention	5.33 ± 1.18[4.00–7.00]	4.500**	**0.000** *
	Control	8.19 ± 0.83[7.00–10.00]		
13	Intervention	5.13 ± 0.99[4.00–7.00]	1.500**	**0.000** *
	Control	8.38 ± 0.89[7.00–10.00]		
14	Intervention	4.73 ± 0.70[4.00–6.00]	0.000**	**0.000** *
	Control	8.31 ± 0.95[7.00–10.00]		
15	Intervention	3.93 ± 0.70[3.00–5.00]	0.000**	**0.000** *
	Control	8.19 ± 1.22[6.00–10.00]		

*P < 0.05;

**Mann Whitney U test. Min = minimum; Max = Maximum; X = mean; SD = standard deviation.

**Table 3:** Comparison of infant stool counts according to the groups (n = 31)

		$\bar{X}$ ± SD [Min-Max]	Test value	P
1	Intervention	1.80 ± 1.15[1–5]	82.000**	0.140
	Control	1.19 ± 0.40[1–2]		
2	Intervention	2.13 ± 1.81[0–7]	61.500**	**0.019** *
	Control	0.81 ± 0.83[0–2]		
3	Intervention	2.00 ± 1.36[0–4]	64.00**	**0.027** *
	Control	0.88 ± 0.62[0–2]		
4	Intervention	2.00 ± 1.85[0–8]	75.500**	0.078
	Control	1.06 ± 0.85[0–3]		
5	Intervention	1.93 ± 1.83[0–7]	99.000**	0.423
	Control	1.19 ± 0.75[0–3]		
6	Intervention	1.73 ± 1.49[0–6]	66.500**	**0.033** *
	Control	0.75 ± 0.77[0–2]		
7	Intervention	1.93 ± 1.39[1–5]	80.500**	0.119
	Control	1.13 ± 0.89[0–3]		
8	Intervention	2.13 ± 1.46[0–5]	58.000**	**0.014** *
	Control	0.88 ± 0.96[0–3]		
9	Intervention	2.20 ± 1.61[0–6]	75.500**	0.078
	Control	1.19 ± 0.75[0–3]		
10	Intervention	1.93 ± 1.22[1–5]	63.000**	**0.024** *
	Control	0.94 ± 0.68[0–2]		
11	Intervention	1.87 ± 1.41[0–5]	86.500**	0.188
	Control	1.13 ± 0.81[0–3]		
12	Intervention	2.07 ± 1.53[0–5]	77.000**	0.093
	Control	1.13 ± 0.96[0–3]		
13	Intervention	2.07 ± 1.53[0–5]	75.000**	0.078
	Control	1.06 ± 0.77[0–3]		
14	Intervention	1.80 ± 1.32[0–5]	66.500**	**0.033** *
	Control	0.81 ± 0.66[0–2]		
15	Intervention	1.93 ± 1.28[1–5]	83.500**	0.151
	Control	1.19 ± 0.66[0–3]		

*P < 0.05;

**Mann Whitney U test. Min = minimum; Max = Maximum; X < in = mean; SD = standard deviation.

The weights of the babies in the probiotics and control groups were compared. Table 4 shows a statistically significant difference in the sixth-month weight of the babies between the groups (P < 0.05). The babies in the probiotics group weighed more at six months than did the babies in the control group. However, there was no statistically significant difference in the height and head circumference of the infants (P > 0.05). The results showed a statistically significant difference between the groups in the nutritional status of the infants at the third month, sixth month, and time of initiation of additional food (P < 0.05) (Table 5).

**Table 4:** Comparison of 6-month-old weight between infants in the probiotics and control group (n = 31)

		Min	Max	Median	$\bar{X}$	SD	Test value	P
Birth weight	Probiotics	2755.00	3790.00	3115.00	3182.00	280.95	-0.706**	0.486
	Control	2250.00	4015.00	3382.50	3293.44	547.24		
1^st^ month	Probiotics	3300.00	5000.00	4100.00	4089.67	501.53	-0.278**	0.783
	Control	3400.00	5200.00	4200.00	4139.38	492.62		
4^th^ month	Probiotics	5280.00	7200.00	6450.00	6383.33	456.41	1.746**	0.091
	Control	4800.00	7420.00	5925.00	6008.13	705.01		
6^th^ month	Probiotics	6450.00	8800.00	7850.00	7699.33	651.15	50.500***	**0.005** *
	Control	1050.00	8800.00	6800.00	6680.63	1675.19		
Birth Size Measure	Probiotics	46.00	52.00	51.00	50.27	1.87	-0.371**	0.713
	Control	47.00	55.00	50.00	50.56	2.50		
1st month	Probiotics	50.00	56.00	54.00	53.33	2.09	0.098**	0.923
	Control	49.00	58.00	53.00	53.25	2.59		
4^th^ month	Probiotics	59.00	69.00	65.00	63.87	2.95	1.798**	0.083
	Control	57.00	68.00	62.00	61.88	3.20		
6^th^ month	Probiotics	64.00	73.00	71.00	69.93	2.76	1.395**	0.174
	Control	64.00	74.00	69.00	68.56	2.71		
Head Circumference on Birth	Probiotics	33.00	36.00	35.00	34.87	0.64	114.500**	0.830
	Control	33.00	37.00	35.00	34.88	0.89		
1^st^ month	Probiotics	35.00	38.00	36.00	36.43	0.86	120.000**	1.000
	Control	35.00	38.00	36.50	36.44	0.81		
4^th^ month	Probiotics	38.00	40.00	39.00	38.60	0.63	99.000**	0.423
	Control	38.00	40.00	39.00	38.81	0.66		
6^th^ month	Probiotics	39.00	43.00	41.00	40.93	1.10	113.000**	0.800
	Control	39.00	42.00	41.00	41.00	0.89		

*P < 0.05;

**Independent t-test;

***Mann Whitney U test; Min = minimum; Max = Maximum; X < in = mean; SD = standard deviation.

**Table 5:** Comparison of feeding status between infants in the probiotics and control groups by month (n = 31)

Variables		Probiotics		Control		Test Value	P
		n	%	n	%		
4^th^ month nutritional status	Breast milk only	14	93.3	9	56.3	5.727*	**0.044** *
	Breast milk + formula	1	6.7	5	31.2		
	Just food	0	0.0	2	12.5		
6^th^ month nutritional status	Breast milk only	1	6.7	0	0.0	7.642**	**0.035** *
	Just food	0	0.0	1	6.2		
	Breast milk + additional food	14	93.3	10	62.5		
	Additional food only	0	0.0	5	31.3		
Cessation of breastfeeding	Yes	2	13.3	6	37.5	2.362**	0.220
	No	13	86.7	10	62.5		
Time of initiation of additional food	4^th^ month	0	0.0	3	18.8	8.471**	**0.018** *
	5^th^ month	4	26.7	9	56.2		
	6^th^ month	10	66.6	4	25.0		
	7^th^ month	1	6.7	0	0.0		
**Sum**		**15**	**100.0**	**16**	**100.0**		

*P < 0.05;

** Chi square analysis.

## DISCUSSION

Infantile colic has a multifactorial etiology, and the sociodemographic and obstetric conditions of the parents, especially the mother, are factors related to the etiology.^8,12,13^ In this section, the sociodemographic and obstetric data of mothers, crying intensity of the baby, frequency of stools, anthropometric measurements, and breastfeeding status are discussed according to the probiotics and control groups and compared with the literature.

In this study, the mean age of the mothers was 25.58 ± 5.04 (Table 1), and approximately half of the mothers (51.61%) whose infants had colic were over the age of 25 years. There was no significant difference between the groups (P = 0.853). In a study of 1955 mothers in whom infantile colic, fetal growth, and other potential risk factors were evaluated, the risk of infantile colic increased as the age of the mother increased.^14^ Several studies have shown high incidence rates of colic in infants of mothers over the age of 30 years.^15-18^ The findings of the current study are in line with those in the literature.

Infantile colic is a behavioral neonatal problem manifested as crying bouts.^19^ Crying time is also an important parameter in the evaluation of the effectiveness of the treatment methods for infantile colic.^20-22^ In the present study, when the crying times of the babies in the probiotics and control groups were compared, a significant difference was found between the groups (P = 0.000). Although the difference in crying intensity between the groups became significant from day 4, the largest difference was observed on day 15. At the end of the fifteenth day, there was an approximately 80% improvement in crying intensity in the infants in the probiotics group, while there was a 12.5% improvement in the control group (Table 2 and Table 3). This finding suggests that the probiotic product was more effective at reducing crying time in infants with colic. In a systematic review evaluating seven randomized controlled trials, probiotics intake was associated with treatment success and reduced crying time by approximately 50 minutes per day when compared with placebo intake.^23^

In recent literature, the presence of anthropometric parameter disorders that an infant cannot develop without any health problems has been added to the diagnostic criteria for infantile colic.^24,25^ In our study, there was a statistically significant difference in the sixth-month weight of the babies between the groups. The sixth-month weight of the babies in the probiotics group was higher than that of the babies in the control group, and there was no significant difference between the height and head measurements of the babies (Table 4). In one study, the growth curves of infants with colic who received probiotic products and formula were evaluated, and it was found that the growth curves of formula-fed infants were low, and the babies were affected.^11^ In a study conducted by Costro-Rodriguez et al.,^26^ there was no significant difference in weight, height, and head measurements when the nutrition of infants with gastrointestinal problems (the most commonly reported gas complaint) was compared with that of the control group. In the same study, the infant growth outcomes in both formula groups were similar to those of breastfed infants in the reference group. In a study conducted by Szajewska and Drly, there was no significant difference in weight between the probiotic-supplemented and non-reinforced (control) groups.^27^ Similarly, the mean changes in height, head circumference, and body mass index were not significantly different between the groups. Our findings are consistent with those of previous studies showing that formulas containing specific prebiotic mixtures and/or fermented formulas are well tolerated and promote adequate infant growth.^27,28^

Excessive crying, which is the most prominent feature of infantile colic, can cause mothers to feel inadequate, decrease breast milk intake, and increase formula use and early transition to additional food.^29,30^ All mothers included in our study fed their babies more than 50% breast milk daily. In the first month, four (26.4%) mothers in the control group compared with those in the probiotics group were giving breast milk and formula to their babies. By the sixth month, 31.3% of the mothers in the control group had stopped breastfeeding (Table 5). In one study, the main reason mothers found their milk to be insufficient was that their babies were crying, causing them to feed their babies formula.^31^ Another study found that frequent crying bouts of infants raised anxiety in mothers about adequate milk intake and were the most important factor that caused mothers to use supplemental products with breast milk.^32^ Contrary to the literature, a study showed no relationship between infantile colic and breastfeeding duration and insufficiency.^33^ Moreover, gastrointestinal discomfort in infants also leads to changes, especially in the transition from breastfeeding to bottle feeding.^11^ In our study, it was found that the babies in the control group (75%) used more pacifiers (40%) than did the babies in the probiotics group. This shows that mothers turn to the use of pacifiers/bottles to reduce the severity of crying. In most cases in one study, mothers decided to stop breastfeeding before seeking medical attention to alleviate gastrointestinal discomfort, and in the same study, reduced diversity and stability of the gut microbiota was associated with the onset of infantile colic.^11^

## CONCLUSION

Infants treated with Actiregularis through their mother’s diet for 15 days showed a decrease in the frequency and intensity of crying, and this had a positive effect on breastfeeding outcomes and anthropometric measurements.
